# Data Collection for Mental Health Studies Through Digital Platforms: Requirements and Design of a Prototype

**DOI:** 10.2196/resprot.6919

**Published:** 2017-06-09

**Authors:** Talayeh Aledavood, Ana Maria Triana Hoyos, Tuomas Alakörkkö, Kimmo Kaski, Jari Saramäki, Erkki Isometsä, Richard K Darst

**Affiliations:** ^1^ Department of Computer Science Aalto University Espoo Finland; ^2^ Department of Psychiatry University of Helsinki and Helsinki University Hospital Helsinki Finland

**Keywords:** data collection framework, mental health, digital phenotyping, big data

## Abstract

**Background:**

Mental and behavioral disorders are the main cause of disability worldwide. However, their diagnosis is challenging due to a lack of reliable biomarkers; current detection is based on structured clinical interviews which can be biased by the patient’s recall ability, affective state, changing in temporal frames, etc. While digital platforms have been introduced as a possible solution to this complex problem, there is little evidence on the extent of usability and usefulness of these platforms. Therefore, more studies where digital data is collected in larger scales are needed to collect scientific evidence on the capacities of these platforms. Most of the existing platforms for digital psychiatry studies are designed as monolithic systems for a certain type of study; publications from these studies focus on their results, rather than the design features of the data collection platform. Inevitably, more tools and platforms will emerge in the near future to fulfill the need for digital data collection for psychiatry. Currently little knowledge is available from existing digital platforms for future data collection platforms to build upon.

**Objective:**

The objective of this work was to identify the most important features for designing a digital platform for data collection for mental health studies, and to demonstrate a prototype platform that we built based on these design features.

**Methods:**

We worked closely in a multidisciplinary collaboration with psychiatrists, software developers, and data scientists and identified the key features which could guarantee short-term and long-term stability and usefulness of the platform from the designing stage to data collection and analysis of collected data.

**Results:**

The key design features that we identified were flexibility of access control, flexibility of data sources, and first-order privacy protection. We also designed the prototype platform Non-Intrusive Individual Monitoring Architecture (Niima), where we implemented these key design features. We described why each of these features are important for digital data collection for psychiatry, gave examples of projects where Niima was used or is going to be used in the future, and demonstrated how incorporating these design principles opens new possibilities for studies.

**Conclusions:**

The new methods of digital psychiatry are still immature and need further research. The design features we suggested are a first step to design platforms which can adapt to the upcoming requirements of digital psychiatry.

## Introduction

Mental and behavioral disorders are the main source of human disability worldwide [1]. Together with neurological disorders, they account for more than 10% of the global burden of disease, exceeding the load of both cardiovascular diseases and cancer [2,3]. In addition to high amounts of years lived with disability due to mental disorders, these illnesses are also one of the substantial causes of death worldwide [4,5].

Mental disorders are challenging to detect and diagnose, in part due to lack of reliable biomarkers [6], and as such, their treatment is a labor-intensive process. Psychiatric diagnoses are typically based on structured clinical interviews that rely on patients’ conscious recall and ability to reflect on past events, thoughts, moods and behavior [7]. However, retrospective recall of variations in the patient’s affective state is inaccurate, particularly if symptomatic variations take place within a temporal frame of hours or days [8,9]. This reduces the accuracy not only in diagnostic evaluations, but also in treatment responses. Besides symptoms, personal, behavioral, and social patterns are also important cues for understanding a patient’s state. Thus, there is a genuine need for conclusive markers.

Because of the increase in use of modern technologies in recent decades, such technologies can collect vast amount of high-quality data on an individual’s daily life and behavior, which are not affected by recall biases [10]. These technologies and the data they produce are promising both in psychiatric research and the clinical domain [11].

The use of technology in psychiatry dates back to early 90’s [12], but recently some studies have used more modern digital platforms to actively and passively collect data from patients, to investigate markers, or to predict new episodes of disorders. Most of these studies focus on schizophrenia and bipolar disorder, such as MONARCA [13], CONBRIO [14], FOCUS [15], and Beiwe [16]. Moreover, most of them have shown promising primary results [16-19]. However, with the high rate of production of new tools, especially mobile phone apps designed to aid patients with mental disorders, the evidence behind the usefulness of these tools is still scant [11,20].

It is clear that more studies are needed to collect large amounts of digital data from patients in order to identify and validate the most useful types of data and to provide evidence for clinical use of digital technology in psychiatry. To date, most studies which collect digital data from patients have used monolithic systems designed specifically for that study. Consequently, the majority of the literature is focused on the results and less attention has been paid to features of the data collection platforms. Inevitably new digital data platforms will emerge in the near future that address the need for large-scale data collection for psychiatric research. Therefore, it is important that research protocols and features of these platforms be shared among researchers in this field, in addition to the results that these platforms produce.

Working in a multidisciplinary group composed of psychiatrists, system designers, and data scientists, we identified the following main features we believe emerging data collection systems for psychiatric research must have: (1) flexibility of access control, to have more simultaneous interdependent studies of the same participants and more control over data mixing; (2) flexibility of data sources, to provide an automatic and systemic linking of diverse sources at minimal upfront cost; and (3) first-order privacy protection, to guarantee the data is being used as the participants consent. The first and second features will give researchers in the field of psychiatry the possibility to easily design studies (even multiple parallel studies) and identify the devices and digital sensors that are most helpful and data from them can be translated to clinically relevant measures. The first feature will also provide data scientists maximum flexibility when analyzing the data, without being restricted by the initial design. The third feature guarantees privacy for patients and will also help to protect researchers against accidental breaches of privacy regulations. Most of these features can be generalized to any digital data collection study; however, because there have not been many studies in the field of digital psychiatry, flexibility both in terms of access control and data sources becomes ever so important. In addition, extra sensitivity of patients’ data calls for more severe measures of privacy. While these features can always be implemented after the fact, top-level consideration makes for the most efficient and secure method of working.

In addition to the identified features, we also presented the Non-Intrusive Individual Monitoring Architecture (Niima) platform designed to meet these 3 key points, allowing fine-grained support for multiple, longitudinal, overlapping studies. Niima integrates various existing data sources powerfully and with flexibility to achieve new data mixing approaches while keeping privacy among different overlapping studies. Niima can be used for randomized studies with patients with mental disorders and healthy controls, or even for studies with general population cohorts where studying the behavior and activity of participants is desired. Data from multiple sources coming from each participant is securely linked to their account on the Niima platform and can later be anonymously accessed by the researchers. Niima ensures the privacy in each part of the data collection, transfer, and analysis processes. Using Niima, researchers can add new sensors to the study during its course without interfering with the data collection already happening or even run multiple studies with overlapping or independent sets of participants simultaneously. Niima also envisions “independent users” who get access to their own data. These users are pilot testers of each study setup that provide feedback to researchers and help optimize the setup.

## Methods

In this section the background for each of the key design principles and how they could be implemented are described.

### Flexibility of Access Control

#### Motivation

Research has advanced to the point where simple studies with human participants are routine. In these studies, data are collected and analyzed to answer the specific project questions. In the context of mental health, the longer people can be observed the better able we are to find clinically relevant variables. However, because using big data in psychiatry is relatively new, there is still little known about what these clinically relevant variables are and how they might be different from one disorder to another. It is now required that we “ scale out”: this does not mean to scale to more data or more participants as this type of scalability is relatively straightforward with modern big data tools. Rather, we mean scalability to longer time periods, more simultaneous data sources, more simultaneous interdependent studies of the same people, more remixing of data, etc. This will give researchers the possibility to validate their findings and apply them in the clinical domain. As an example, one might want to conduct repeated data collection experiments with the same cohort of mental-health patients, say, initially using phone tracking apps, so that additional sources of data—bed sensors or activity trackers—are incorporated as soon as their value to the researchers becomes apparent.

In order to achieve its goals, a data collection system must adopt a good model of users, data sources, studies, and the flow of data between these. If it does not, it may be limited to the “one pot” model of data collection, without the ability to manage the process and data flows. The data model described here allows us to achieve our other goals of flexibility of data sources and privacy easily.

#### Implementation

##### Access Control

**Figure 1:**
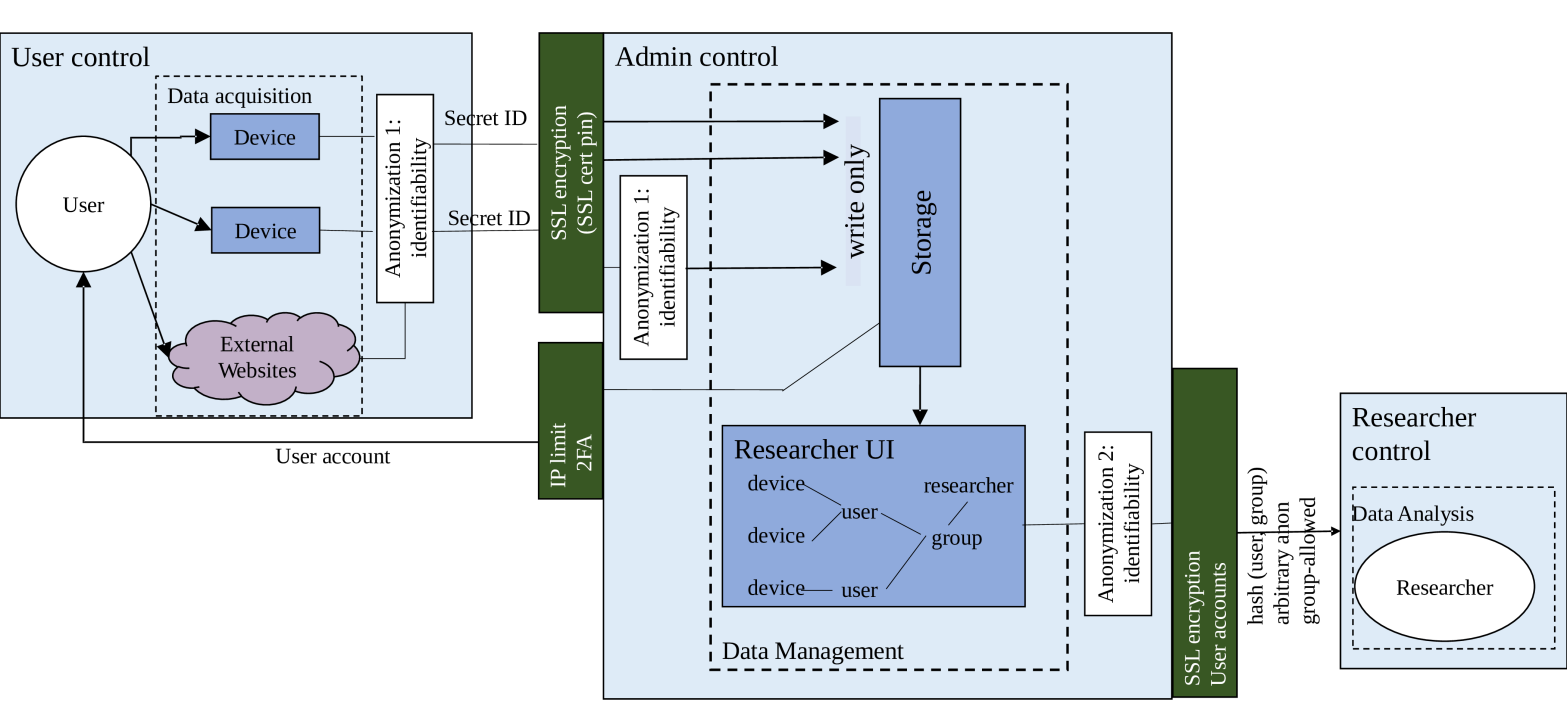
Outline of the Niima privacy model. The strict separation of roles user, researcher, and administrator provides the basis of all work. Data anonymization can be performed both before data is received and again before it is provided to researchers. The study-user-device model provides for fine-grained access control.

Niima implements 3 types of users: administrators, researchers, and users (participants) (Figure 1). Administrators have the ability to set up the study parameters, but do not access raw data for research purposes. The administrators may be system-wide, or per-study, depending on the level of independent supervision needed. The managers (a type of researcher) set up the study and have access to the creation of users and devices. Managers and other researchers only have access to data after it has been processed for privacy. The participants or users are the people who provide data. This 3-tier system provides the basis of our privacy system. While this seems obvious, it is important to properly plan and design things in advance. Once a proper role system exists, we can add rules which further improve data protection by limiting access to different people. For example, one could separate those who interact with the participants from those who access the data, providing further privacy to participants. If a proper role system does not exist, then implementing access control becomes difficult and error-prone.

With Niima, across different studies, users will preserve the same user account, while their role might change from one study to another. In addition, they can be simultaneously part of different studies while having a different role in each. It is even possible for one user to have more than one role within a single study (Table 1).

**Table 1:** The role system structure of Niima.

Actions	Role				
	Administrator	Manager	Researcher	Participant	Independent user
Has user account	Yes	Yes	Yes	Yes	Yes
Configures study	Yes	No	No	No	No
Adds/removes users within study	No	Yes	No	No	No
Views raw data	No	No	No	No	Yes (only own)
Enrolls participants and configures their devices	No	Yes	No	No	No
Has access to data after converters	No	No	Yes	No	Yes
Gets access to own user account	Yes	Yes	Yes	No	Yes

An individual’s data is stored connected to a user account on the server. A user account is separate from any particular study. Users may be a part of none, one, or more studies. A user not associated with any study is defined as an “independent user” and is discussed below.

##### Study Model

Each study contains various metadata, which provides fine-grained access control. First, it contains participants and researchers. Data from participants is made available to the researchers. A start and end date defines the available data range, which is important so that more limited data access may exist even while data is being collected longitudinally. The study also defines “converters,” which specifies what data is available to the researcher. Because the converters are defined per-study, each study’s researchers will get only the data necessary for their purposes, even if data from participants is being collected for other studies.

Each participant may have multiple devices of any type, so, for example, if a user has 2 phones, data can be collected from both of them. A “device” only consists of a device identification (ID) and a place to store data under that ID. This is what allows us to easily scale the data collection task to any number of data sources per person.

Independent users (not part of any study) can view all of their own data, including the raw data. One reason creating independent users was that in contrast to classical clinical studies, studies which use digital platforms require pilot participants and testers. Having independent users makes it possible for researchers to test the system with these users (who preferably have technical knowledge or study-specific knowledge) and can give good feedback about the study design and setup.

Transparency—the right to inspect one’s own data—is an important principle of personal data processing [21]. Our system has the possibility of transparency by allowing user accounts for participants, which can be used to inspect their own data like independent users can. However, where this is not desired (eg, clinical studies), it is not required. Because of this design, our system is equally usable as a public service. This can be used to attract volunteers as part of studies. Thinking about both the within-studies and independent use cases together forces us to design a system with a focus on individual rights, making privacy natural.

In our design thus far, independent users are given full access to their own raw data in addition to converters which make the data easy to use. Studies are limited in the converters they provide, to only allow required data through.

Separating users and studies and raw/processed data allows new possibilities and stronger privacy-preserving properties (see below). First, converters allow a much more fine-grained approach to privacy protection. Second, this is set up as a third-party anonymization service, which mediates between participants and researchers. With this system, a third party can provide privacy supervision and can help to ensure ethical supervision is being fulfilled. It can also allow longitudinal data collection with various overlapping short-term studies and even a safe way to collect more data than at first deems necessary. Finally, the introduction of independent users allows for a more participatory model of science, which is especially important when dealing with personal data.

### Flexibility of Data Sources

#### Motivation

To find clinically relevant variables, we should not only look at different data coming from various sources in a certain device (eg different data streams produced by mobile phones), but also at other wearable and consumer devices that have become ubiquitous and can provide rich behavioral data (ie, activity trackers, ballistocardiogaphic bed sensors, and other types of devices) that are available to consumers [22]. It is important that data from these devices be studied and validated. Given the combination of many possible data sources and lack of certainty of the most useful and clinically relevant data, any data collection system must be flexible as to the data it collects. Another reason why it is important to have the possibility of collecting data from multiple devices for the same individual at the same time is that mental disorders and chronic physical illness often appear as comorbid conditions [23]. As such, it would be too simplistic to treat comorbid conditions separately and to follow their trajectories individually [24]. Therefore, there is a need for changing the way psychiatric care is delivered that addresses this complexity [23]. There are many frameworks and mobile phone apps that help with monitoring chronic physical illnesses [25,26]. Having the possibility of collecting data on physical and mental comorbidities at the same time in future studies, would provide valuable insights for better understanding these comorbidities and providing better solutions for them.

#### Implementation

To be a viable research tool, a system must be able to quickly adapt to new sources of data. Our system is designed from the ground up to integrate many sources of data. The goal is to be able to integrate any new data source with minimal upfront cost. As new data sources are identified, we can quickly integrate them because of the flexibility of our data model.

Our platform adopts the “lambda architecture” of data systems [27]. In this architecture, all incoming data is saved raw and not modified. Data interpretation and processing is performed as a second stage, which can be repeated and improved as needed with no risk of data loss. Currently, real-time access to data is not a priority of Niima, but should such access be needed, a third stage can store the processed data in secondary databases suitable for real-time querying and operations.

When data arrives in lambda architecture, it is stored raw (usually the raw text is received) and represented in whatever the original format was. This lowest common denominator format can work and is efficient enough for any device with modern storage and transparent compression. This means that data can be inserted in an efficient write-only operation, which also improves security. The flexibility and simplicity, and thus reliability, of this system more than makes up for any inefficiencies. We can easily accept data from any type of device, even devices with very limited programmability. Data is identified by a secret token when it is received, which is directly used to store the data. Data storage is stored only indexed by (device ID, timestamp), which allows us to use modern databases that scale to huge amounts of data.

The bases of the extraction stage are converters that convert raw data into structured, table-based formats. For example, a mobile phone device could have a converter that translates the raw text data about communication activities to call records, rehashing numbers and removing self-calls. Another example is converting detailed Global Positioning System (GPS) location data to quantities such as the average amount of movement per hour, in order to better preserve privacy. Each device type per study can have different converters ensuring that the data provided is specific and minimal.

One important benefit of this system is that the interpretation of the data is delayed to the conversion phase. The marginal cost of adding a device is very small; all that is needed is some interface to receive data and store it raw. All processing can be delayed until the relevant contents of the data are identified. This is especially important from a privacy perspective such that privacy and anonymization decisions do not have to be made immediately, but can be made (1) after the exact data needs are identified, (2) per-study (data minimization), (3) after unforeseen privacy risks are handled, and (4) in a fashion that can be minimal at first and improved over time.

This system also provides a decoupling between data source and usage. In a large study, data may come from devices that are not under the researcher’s direct control. This may happen if, for example, our data is collected from an external service. It could also happen if a study lasts a long time and data sources need to be updated. It is easier to maintain a flexible data model than manage migrations. When all data is stored raw, it means that all but the largest of changes have no impact on receiving data. By versioning or simply examining the data received, the second-stage converters can decide the proper way to process it at some later time. This greatly simplifies the long-term maintenance of studies and allows us to maintain higher quality and more adaptable data collection for a longer time with fewer resources.

To demonstrate this system, we integrated a wide variety of data sources into our prototype system: 3 Android and 1 iOS app [28-30], an Internet of Things (IoT) device [22], server-side surveys, manual upload, actigraphs, and social media. The mobile phone apps send data using various application programming interfaces (APIs), and raw data is stored as the raw Javascript Object Notation (JSON). The IoT device is a Murata bed sensor [22] that also sends data via a Web API, but our server’s endpoint must examine the data to determine the proper device ID. We have implemented server-side surveys, where participants can be asked questions and data is saved in JSON. For social media, our server interfaces using the public APIs. Finally, our system can be used for any other data. For example, we integrated Phillips Actiwatches (actigraphy devices). These were completely non-networked devices. Data was extracted from them with their existing software and then uploads were done manually, specifying only the proper device ID to link data with the user’s profile. From that point, our system of converters and privacy tools took over.

With this system, we can effortlessly collect and merge data from almost any device. This forms the core of a vision where data may freely flow between different apps and platforms, as opposed to just from one data source to its intended collection server. The major lessons for developers are (1) separate data collection from data use (upload) via a simple API expecting that others will use it in other contexts, and (2) provide a method to specify the upload server address.

### Privacy Protection

#### Motivation

Personal privacy is a critical part of research. It is especially important in health-related fields, where ethics and law are strict in their standards. Confidentiality is the cornerstone of all psychiatric patient evaluation or treatment. However, many existing data collection frameworks adopt a limited privacy model. These studies generally collect data with a “one pot” model: data is collected and goes straight to researchers, stripping of direct identifiers. However, privacy is much more than this, it includes purpose limitation, fine-grained access control, tailoring anonymization to the intended purpose, etc [31]. While there are an almost unlimited number of things that could be said about privacy, there are a few important lessons. Some basic principles of ethical data access include (1) consent, meaning a person must approve the way data is used; (2) a participant should have access to their own data for examination; and (3) data minimization [32-34]. The basic solution to privacy in a scientific context is anonymization, the idea that while data may be shared, actual identities are not known and are not to be discoverable.

Unfortunately, this does not provide an actual solution, because instead of some specific criteria, this requires a lack of possibility of identification, which cannot be easily proven for our multidimensional and long-term data. Techniques such as k-anonymity and l-diversity can provide guidance about making data anonymous; however, because our type of longitudinal data is highly multidimensional these techniques do not apply [35,36]. With detailed, multifaceted data on people, it is nearly impossible to prove that a true anonymization has been done without destroying some useful aspect of the data. Thus, our architecture is designed around the principle of defense in depth. First, we do not collect direct identifiers at all, even as raw data. Arguably, this could make our data anonymous according to some interpretations. Second, when data is extracted, it is further anonymized, for example, by rehashing or aggregation. Third, each study can have its own separate anonymization based on a secret seed. This prevents linking data between different studies. Finally, researchers must agree to data protection by an agreement, including conditions of not identifying people. The combination of the above factors can be enough to satisfy the standard of “not reasonably likely” that a person could be identified, which is the most common standard. However, since there is no universal standard, all we can do is provide the tools and allow people to use them as needed.

#### Implementation

##### Identification Versus Linkability

It is important to note the difference between identities and linkability. Privacy risks appear when data can be linked to specified real persons. It reasons that in order to preserve privacy, we must eliminate the ability to link different data together. Removing direct identifiers is the most obvious way of preventing linkability to real persons. However, linking de-identified data to other de-identified data is also a risk, because once more is known it becomes easier to re-identify. With our more flexible data model, we have the possibility for multiple studies with access to different data from the same people. Our system provides tools to remove these possible links as well.

##### Converters

Converters are the basis of our privacy strategy. Each converter applies arbitrary transformation to the data before it is presented to the user. This gives us the ultimate flexibility of managing privacy. Converters do not only directly translate data, but can provide higher level operations such as aggregation. Further, since converters operate right before data is extracted, privacy can be continually improved. It is also possible to begin by releasing the minimal amount of data, and then incrementally release more as it becomes necessary or safe.

##### Hashing

Hashing of identifiers is the anonymization strategy. A one-way hash is a function which, given some data, produces a new meaningless identifier that is a function of that data [37]. These functions have mathematical properties such that the input cannot be derived from the output, which is useful for anonymizing identifiers such as phone numbers while still being able to connect identical events. While it is impossible to derive the input from the output, when there are a limited number of possible inputs, it is possible to test all inputs to determine the output, especially because these functions are designed to be fast. Purposefully slow hashing methods, such as those designed for password hashes, are too slow to apply in bulk and still are designed to allow one to verify a known input. Thus, we hash identifiers on the server using a secret salt, hash(salt+data) [38]. If the salt is long and secret, this allows for both speed and complete security. This is also done as a 2-stage process: first data are normally hashed on devices and a second round is done on the server with the secret salt. This is because a secret salt cannot be transferred to devices. This naturally integrates into the Niima architecture. Further, each study can use a different secret salt, so that the data for each study cannot be linked. Finally, in order to perform a final, absolute, and irreversible anonymization, the data can be exported with a one-time salt that is not stored anywhere.

##### Timestamp Anonymization

A similar process can be used for timestamps. In our data, even a single timestamp can prove to be identifiable. If a person knows that a user performed some action at a certain time (such as making a phone call), then regardless of any other anonymization, if that timestamp is unique in the data, the person can be identified. For larger amounts of data, even knowing times of a small number of events can fingerprint a specific user. Our system passes all timestamps through an arbitrary function, which can be used to apply a fuzzing to adjust timestamps. The exact function and amount of shifting must be decided based on what is necessary for research and anonymization.

##### Institutional Control

Hashing and timestamp fuzzing are only some examples of the types of privacy-preserving transformations made possible by the Niima architecture. Our structure is that of a third-party anonymization service. There is a strict separation of roles between the server administrators (who have technical control of the server) and researchers, and these levels balance between research flexibility and privacy. It is the administrators’ basic responsibility to manage the research consent between participants and researchers and to guide the researchers so that privacy is protected. In the upcoming European General Data Protection Regulation, there is an emphasis placed on privacy by design as well as institutional control [21]. The structure of our system provides a good basis for satisfying both of these criteria.

## Results

In this section, use cases made possible by our work are discussed. As these are not classic studies, they require a very refined model of users and privacy. In the first, a student course was organized, primarily designed for pedagogical purposes. The flexibility of the system allowed the students to have full control over their own data and to adapt as the students proceed. In the second, we use the flexibility of our system to pilot a project.

### Student Project

Collecting data from people is not just useful for scientific studies, but can be useful for pedagogical purposes as well. Working with students also provides an extremely rigorous test of privacy procedures, since students have a right to study without their data being required to be shared. We hosted a course where we provided the basic tools for data collection (Niima) and the students had freedom to choose the data sources and research questions they were interested in. As part of the course, students considered and solved privacy issues related to each of the data streams. Because this was a student project, we could not simply adopt the “one-pot” model of collecting all data and distributing it to everyone.

In the course, the students first collected their own data and independently analyzed it. Our framework’s independent user accounts naturally facilitated this. After a period of considering their own data, the students planed research problems which used everyone’s data. They considered privacy issues and decided what data could be shared, and most importantly, how anonymization could be performed. Their anonymization procedures were implemented into converters for their data sharing groups. In order to protect study rights, data sharing was voluntary, and to ensure there was no possible effect on grading, even instructors did not know who had opted into sharing data. This was handled by anonymous opt-in on the server. Thus, during and after the project, no instructor could even know which students opted for sharing; the only way to guarantee that there was no unconscious bias.

The students used a wide variety of data sources, including mobile phone apps (Purple Robot) [28], surveys, and the Murata Bed Sensor Node [22]. We could quickly adapt to the interests of the students. Partway through the course, some Philips Actiwatch II sensors became available and students decided to augment the data with this. Because of our design, adding these devices was trivial, even though they were non-networked and managed by legacy software. We added a new server device class for the Actiwatches, and students added it to their user accounts. Students provided only the device ID and this was used to register devices so that the instructors did not need to manage or ever record the subject identities themselves. The output data files were uploaded to the server so that students could examine them. After this, the relevant data was identified, converters were written, and the data was made available via a study.

Our system also allowed for incremental sharing. As we stated above, students always anonymously opted in to data sharing by joining a new study. By the end of the course, students were enrolled in different studies, each of which was for sharing a certain type of data. This provided granularity and specificity of purpose. Initially, only certain safe data was shared for a limited purpose of this course. Later on, students could anonymously opt in to sharing data for other purposes, such as donation for follow-up research or even sharing as open data.

The course was a vital pedagogical tool in these students’ data science training. Real-world data and real-world problems in collecting the data are different from what is typically experienced in class. Unlike in structured courses, students experienced the difficulties in data wrangling and cleaning, which is a skill that can only be learned by doing. The Niima architecture was necessary because of the complex nature of the subject-researcher relationship and the fast-moving development of the project. The lessons and tools here also provided valuable lessons for prototyping other studies.

For us, this student project showed how data processing and access could be controlled with our system. Access for the data could be limited for each user type which made preserving privacy possible. Each data source required separate converters for anonymization and preprocessing. After setting up the converters, providing anonymized data and fine-grained access was easy.

### Preparing for a Future Study

We will start using our tools in studies at the Department of Psychiatry of the Helsinki University Central Hospital. In preparation for this project, extensive testing is needed. We would like to get as many testers as possible; however, these testers also need privacy while still closely interacting with the developers. Moreover, because of the clinical implication of the data, privacy is an especially large concern. We divide the researchers into 2 categories: those who interact with participants (managers) and those who have access to the data. This access control was natural given Niima’s design principles. This allows us to tell testers that their privacy is maintained; there is not one single person who can have access to both the identities of the participants and the data about them.

The data will be collected for 1 year uninterruptedly with different devices (mobile app, actigraphy, sleep sensor, experience sampling methods/ecological momentary assessment- (ESM/EMA-) based questionnaires, etc) at different moments in the research. The overlap of devices and change over time are easy to set up in Niima because of its scalability over time, participants, and data mixing. Moreover, Niima allows the grouping of different data sources of individual participants because of its flexibility of data sources, so even if the study requires a second change over its course, it will be possible to achieve and reconfigure easily.

## Discussion

### Principal Findings

It is important to distinguish between biomarkers used as (1) diagnostic tests, and (2) their use as indicators of illness state variations [39]. Our first goal when collecting data from patients with mental disorders should be the latter, because before being able to benefit from them in practice, we need to answer the following crucial questions: (1) to what extent do parameters extracted from passive data covary with clinical states, and (2) how good of an indicator of clinical state can digital tools get? There are some studies that have tried to investigate this with respect to depressive symptoms by collecting data from a general population cohort and have provided a first proof-of-concept [40-42]. However, there is need for clinical trials with enough statistical power to maximize the chance of finding new biomarkers from the data. Even for bipolar disorder there are relatively more studies that have collected and analyzed passive data from patients [43]. More data from different populations are required so that in addition to new findings, previous results of past studies can be validated. In addition, there is a need for developing new methods for learning from such data [44]. These methods can be developed only if we have good data collection tools which can be widely used and if we can guarantee that experts from psychiatry work together with data scientists. Upon developing and validating such methods, if they prove to provide a better understanding of mental disorders, the newly found biomarkers can be used as diagnostic tests. However, real-world performance of a diagnostic test depends on prevalence of illness, thus, utility of any diagnostic tool is context dependent. Clinical trials should be performed as the ultimate utility test, in which patients’ outcomes should be shown to be better using these methods.

Here, we presented features we believe are important for the design of a data collection platform for mental health studies and described a data architecture which allows more and better data to be collected. First, we outlined a model of users and studies that allows for a more sophisticated and flexible process from the perspective of the user. With this, it is natural to be able to run longer and more detailed studies. Second, our method of processing data allows much more flexibility of data sources and new data sources can be added with trivial cost. Combined with the first point, we no longer conceptualize research in terms of studies, but research in terms of people who are thoroughly quantified, where data flows in from sources and out to different users. In order to make this feasible, a model of privacy better than “remove identifiers” is needed. This model is both the third point and an outcome of the first two.

To demonstrate the power of this system, we implemented an initial prototype, Niima, which implemented the principles described above. By using this platform, we were able to engage in studies with more rigorous demands on privacy and flexibility than are possible using existing systems.

In the future, Niima can be used for different types of studies, and while studies with patients with different mental disorders has been one of the main use cases of the framework, it can be used in any kind of study requiring multi-sensor and/or multi-device data collection from human participants. Designing a platform for the original use case (patients with mental disorders) is perhaps the most challenging case of all types of data collection studies with human participants. By designing a system that accommodates the needs of one of the most difficult cases, the system can even more easily be used for behavioral studies for general population cohorts.

### Conclusion

While we hope that technology quickly adapts to the needs of science that is not always the case. In many cases, the limits of technology set the limits of research. The models we propose do not so much represent a revolution in either science or technology independently, but represent new ways of using technology for science. Adopting these models, however, will not be instantaneous. In particular, proper methods will provide an agility for research which does not match the demands of pre-approval for human participant experiments. However, the models we propose are a definite improvement in the protections of the rights of participants, and we can hope the ability of better technology can eventually influence research ethics.

## Abbreviations

API: application programming interface
ID: identification
IoT: Internet of Things
JSON: Javascript Object Notation
Niima: Non-Intrusive Individual Monitoring Architecture
